# Regional variation in low-value musculoskeletal surgery: a nationwide study from the Finnish Care Register

**DOI:** 10.2340/17453674.2024.41930

**Published:** 2024-09-20

**Authors:** Ville PONKILAINEN, Anniina LAUREMA, Ville M MATTILA, Teemu KARJALAINEN

**Affiliations:** 1 Department of Orthopaedics and Traumatology, Tampere University Hospital; 2 Department of Surgery, Mikkeli Central Hospital, Mikkeli; 3 COXA Hospital for Joint Replacement, Tampere; 4 Faculty of Medicine and Life Sciences, University of Tampere, Tampere; 5 Department of Surgery, Central Finland Central Hospital, Jyväskylä, Finland

## Abstract

**Background and purpose:**

Healthcare systems globally are grappling with resource constraints and rising costs. Concerns have been raised about “low-value” care, which consumes healthcare resources without benefiting patients. We aimed to examine regional differences in common low-value musculoskeletal surgeries in Finland and explore explanatory factors behind the variation.

**Methods:**

Using data from the Finnish Care Register for Health Care, surgeries conducted from 2006–2007 compared with 2020–2021 were analyzed across 20 hospital districts. Selected surgeries (acromioplasty, rotator cuff repair, partial meniscectomy, wrist arthroscopy, ankle arthroscopy, and distal radius fracture fixation) were categorized based on NOMESCO procedure codes, and incidence rates in older populations were calculated based on population size derived from Statistics Finland.

**Results:**

We found substantial regional disparities in low-value surgeries. The incidence rates were higher in hospitals with high historical incidence rates and smaller population sizes, suggesting that the uptake of evidence is slower in small non-academic hospitals.

**Conclusion:**

The incidence of low-value surgery is declining but regional differences remain large. It is unlikely that regional variation in disease incidence explains such large variation in low-value surgery. Instead, local treatment culture seems to be the driving force behind low-value surgery, and the practices seem to be more entrenched in small hospitals.

Increasing demand and rising costs are straining healthcare systems worldwide. Multiple global, national, regional, and local factors drive the soaring costs, yet 80% of the total costs are initiated from the decisions physicians make at individual care level [1,2] . One of the recently highlighted reasons for rising costs is “poor” or “low-value” care practices, i.e., treatments that produce little or no benefit to patients or are harmful to patients [2]. For example, in the USA, low-value care is estimated to account for up to 20% of healthcare costs and its impact is constantly increasing [3].

The global and national use of surgical procedures varies widely. Variance in disease prevalence, diagnostic practices, and patients’ expectations explain some of the regional differences. The primary factors behind variation in low-value surgery are the surgeons’ and patients’ perceptions of the efficacy of surgery [2,4]. Recently, many common musculoskeletal surgeries have been proved to have very low effect or even to be ineffective [5-10]. The evidence has been effectively adapted into daily practice in Nordic countries [11-19], yet regional variation has been present [20]. Understanding the factors that contribute to the persistent utilization of low-value care in certain regions, while others abandon inefficient practices, could facilitate the development of efficient de-implementation strategies.

The aim of our study is to describe the regional differences in low-value surgery in Finland from years 2006–2007 compared with 2020–2021 and to explore factors that are associated with the variation in low-value surgery.

## Methods

### Study design and data source

The data for this nationwide retrospective observational register study was obtained from the Finnish Care Register for Health Care, covering data from January 1, 2006 to December 31, 2021. Data on date of surgery, patients’ age, hospital, municipality of residence, primary operation codes, and diagnosis was obtained from the register. It is mandatory to report the surgeries to the register, thus coverage (92–98%) and sensitivity (81–92%) of the register have been shown to be high [21].

The healthcare system in Finland comprises both public and private sectors. Public healthcare services are administered by the Finnish state and are available to all permanent residents of Finland. Private healthcare services are also accessible to all residents, with costs covered by patients themselves, insurance, or private occupational healthcare organizations. Our dataset encompasses all medical procedures conducted in both public and private hospitals within Finland.

The Finnish healthcare system was divided into 20 hospital districts during the study period. Geographical location of hospital was based on the hospital code, which was combined with the data including all hospital districts in Finland. Hospital district data was publicly available from Statistics Finland [22]. For 609 of 170,385 (0.35%) surgeries performed in private hospitals, the hospital district was not provided. For these operations, patients’ home municipality was used to define the hospital district of the surgery. No surgeries were performed in Åland Hospital District, and it was removed from the analysis. The records from Kymenlaakso Hospital District (Kotka and Kouvola hospitals) were lacking year 2020 and 2021, and were therefore removed from the analysis.

### Parameters

Low-value surgical operations were identified based on primary NOMESCO procedure codes (Finnish version) [23] together with ICD-10 diagnosis codes. Included surgeries were acromioplasty (High certainty of evidence, 8 trials: Little or no benefit [7]), rotator cuff repair (Clinically unimportant evidence [6]), partial meniscectomy (High certainty of evidence, 16 trials: Little or no benefit [24]), wrist arthroscopy (No supporting evidence [25]), ankle arthroscopy (No supporting evidence [26]), and distal radius fracture fixation (High certainty of evidence, 12 trials: Clinically unimportant compared with cast in people > 60 [27], evidence is limited only to distal radius fractures with dorsal displacement [Colles]) (Table 1). The age limitations were ≥ 18 years for acromioplasty; > 40 years for partial meniscectomy, wrist arthroscopic debridement, and ankle arthroscopy and > 65 years for rotator cuff repair and distal radius fracture fixation.

**Table 1:** NOMESCO procedure codes, diagnosis codes, age limitations, and certainty of evidence for all evaluated surgeries

Surgery/NOMESCO code	Diagnosis codes	Age, years	Evidence	Certainty of evidence
Acromioplasty NBG10 Acromioplasty NBG15 Acromioplasty, arthroscopic	M* Diseases of the musculoskeletal system and connective tissue.	> 18	Little or no benefit	High, 8 trials [7]
Partial meniscectomy NGD05 Partial excision of meniscus of knee, arthroscopic	S* Injury, poisoning and certain other consequences of external causes.	> 40	Little or no benefit No supporting evidence**^a^**	High, 16 trials [24] NA**^a^** [25]
Wrist arthroscopy NDF25 Operation for osteochondritis of joint of wrist, arthroscopic	T9* Sequelae of injuries, of poisoning and of other consequences of external causes		No supporting evidence**^a^**	NA**^a^** [26]
Ankle arthroscopy NHA30 Exploration of joint of ankleor foot, arthroscopic NHF* Operations on synovia and joint surfaces of ankle and foot				
Rotator cuff repair NBL00 Suture or reinsertion of rotator cuff NBL05 Arthroscopic suture or reinsertion of rotator cuff			Clinically unimportant benefit	NA**^a^** [6]
Distal radius fracture fixation NDJ62 Internal fixation of fracture of wrist or hand using plate and screws NCJ62 Internal fixation of fracture of forearm using plate	S52.5 Fracture of lower end of radius S52.4 Fracture of shafts of both ulna and radius	> 65	Clinically unimportant compared with cast in people > 60**^b^**	High, 12 trials [27]

a No trials comparing surgery and nonoperative treatment or placebo.

b Evidence is limited only to distal radius fractures with dorsal displacement (Colles).

NA = not applicable.

### Statistics

The annual incidences (per 100,000 person-years) were calculated based on the mean adult population (age ≥ 18 years) of each Finnish hospital district obtained from the publicly available register at Statistics Finland [28]. The incidences were calculated for each surgical procedure using the population with similar age limitations as for the procedure (see Table 1). Poisson regression with exact method was used to compute the 95% confidence intervals (CI) for the incidence rates [29]. We calculated the mean incidence for 2006–2007 for historical reference and for 2020–2021 to present current practice. The reference years were chosen based on previous studies [14,30,31] showing that the incidence of arthroscopies and rotator cuff surgery were highest in Finland between the years 2006 and 2007.

To explore which factors could explain variance in the incidence between the hospital districts, we performed multiple Poisson regression analyses: (i) association between incidence of low-value surgery in years 2020–2021 and historical reference (years 2006–2007) adjusted with mean district population size; (ii) association between the incidence in public and private hospitals, adjusted by the reference incidence (2006–2007); (iii) association between the incidence in public hospitals and mean district population size, adjusted with the incidence of historical reference (2006–2007). The mean population size was interpreted in regression models as 100,000 residents. The results of the regression analysis were interpreted as regression coefficient β with 95% CI. The residuals of each Poisson regression model were normally distributed, confirmed with Q–Q plot and the Shapiro–Wilk normality test.

A data set containing a map of Finland with hospital district boundaries were obtained from publicly available R package SHP2019 [32]. All analyses were performed using Mac R version 4.3.2 (R Foundation for Statistical Computing, Vienna, Austria). Data cleaning was performed using the tidyverse (https://www.tidyverse.org/) package. Function epi.conf from R package epiR was used for the incidence rate calculations.

### Ethics, registration, data sharing plan, funding, and disclosures

This study was approved by the Finnish Institute for Health and Welfare (THL/1800/5.05.00/2019). Informed consent was waived due to the retrospective and de-identified nature of the data. The data was sourced from the Finnish Care Register for Health Care, ensuring patient confidentiality with data protection regulations. All procedures followed ethical standards in accordance with the Declaration of Helsinki. The data used in this study cannot be shared publicly due to restrictions imposed by Finnish data protection legislation. Access to the data is limited to authorized personnel and governed by strict data privacy regulations to ensure the confidentiality and privacy of the patients involved. This study has not received any external financial support. None of the authors have any conflicts of interests to declare. Complete disclosure of interest forms according to ICMJE are available on the article page, doi: 10.2340/17453674.2024.41930

## Results

**Figure 1:**
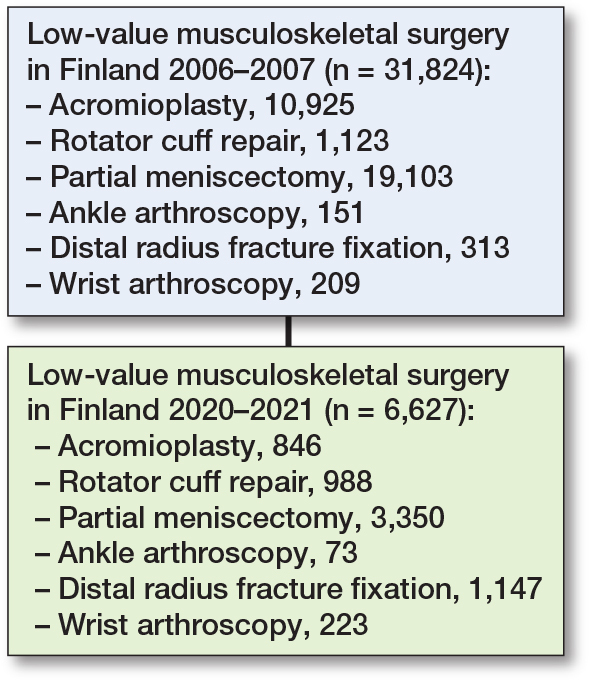
Flowchart of procedures 2006–2007 and 2020–2021.

The total number of low-value surgeries declined from 31,824 in 2006–2007 to 6,627 (–79%) in 2020–2021 (Figure 1). Within the 20 hospital districts, the median incidence was 15 per 10^5^ person-years (range 7–40, IQR 12–16).

**Figure 2:**
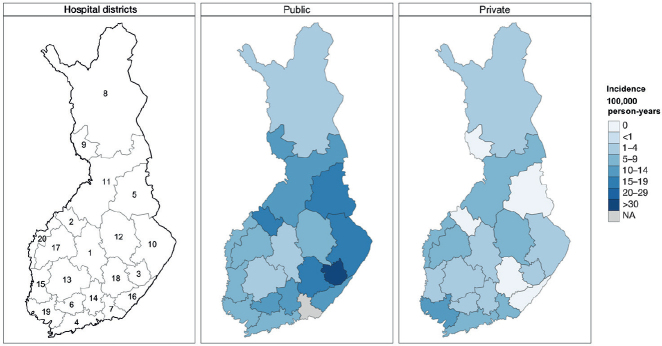
Total annual incidence (per 105 person-years) of low-value surgeries by hospital districts in Finland in 2020–2021, divided into public and private sectors. For name of hospital districts, see Table 2.

In public hospitals, the total incidence of low-value surgeries ranged between 3 and 35 per 10^5^ person-years, while in private hospitals, the incidence ranged between 2 and 13 (Figure 2, Table 2). In Central Ostrobothnia, Kainuu, Länsi Pohja, South Karelia, or South Savo Hospital Districts no low-value surgeries were performed in private hospitals likely due to no private service providers in the districts.

**Table 2:** Incidence of low-value care based on hospital district, divided into years 2006–2007 and 2020–2021 by hospital type (private vs public)

Hospital district	Private hospitals		Change (%)	Public hospitals		Change (%)
	2006–2007	2020–2021		2006–2007	2020–2021	
1: Central Finland	7.3 (4.0–12)	2.3 (0.73–5.5)	–68	58 (48–70)	4.2 (1.9–8.1)	–93
2: Central Ostrobothnia	–	–	–	89 (67–120)	19 (9.9–34)	–78
3: East Savo	–	4.3 (0.29–19)	–	120 (85–160)	35 (18–62)	–70
4: Helsinki and Uusimaa	19 (17–22)	7.3 (6–8.9)	–62	37 (34–41)	5.0 (3.9–6.3)	–87
5: Kainuu	–	–	–	59 (42–81)	15 (7.0–29)	–74
6: Kanta–Häme	9.5 (5.0–16)	4.0 (1.4–9)	–58	100 (88–120)	12 (7.2–20)	–88
7: Kymenlaakso	1.6 (0.25–5.4)	7.5 (3.6–14)	360	42 (32–53)	NA	–
8: Lapland	–	3.2 (0.69–9.2)	–	70 (54–89)	4.8 (1.5–12)	–93
9: Länsi Pohja	–	–	–	84 (61–110)	14 (5.4–29)	–83
10: North Karelia	14 (8.6–22)	2.3 (0.48–6.5)	–84	61 (49–76)	18 (11–26)	–71
11: North Ostrobothnia	21 (16–27)	5.4 (3.2–8.7)	–74	42 (35–50)	10 (7.0–14)	–76
12: North Savo	8.3 (4.8–13)	6.5 (3.4–11)	–22	73 (61–85)	7.3 (4.1–12)	–90
13: Pirkanmaa	28 (23–34)	5.0 (3.1–7.5)	–82	49 (42–56)	3.0 (1.6–5.1)	–94
14: Päijät-Häme	8.6 (4.7–14)	3.3 (1.2–7.3)	–61	49 (39–61)	12 (7.8–19)	–75
15: Satakunta	11 (7.0–17)	2.1 (0.52–5.5)	–82	44 (35–54)	7.0 (3.7–12)	–84
16: South Karelia	15 (8.7–24)	–	–100	68 (53–85)	13 (6.8–21)	–81
17: South Ostrobothnia	7.6 (3.9–13)	5.7 (2.6–11)	–24	60 (48–73)	9.2 (5.0–15)	–85
18: South Savo	0.13 (0.00–4.5)	–	–100	69 (53–89)	16 (8.7–28)	–77
19: Southwest Finland	25 (21–31)	13 (10–17)	–48	68 (60–77)	5.1 (3.1–7.9)	–92
20: Vaasa	8.1 (3.9–15)	7.6 (3.7–14)	–5.9	27 (19–38)	8.8 (4.5–15)	–68

NA = not available.

**Figure 3:**
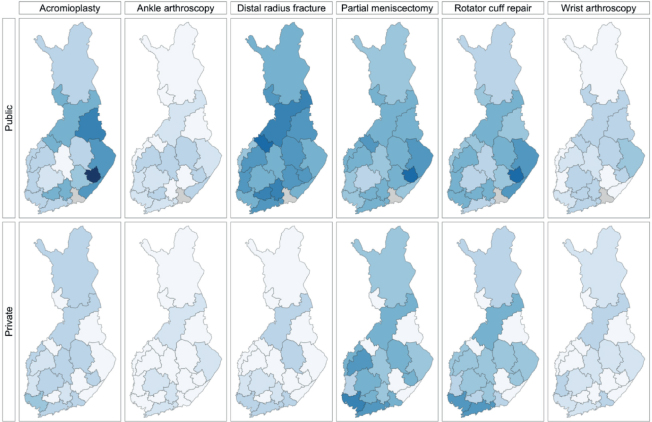
Total incidence (per 105 person-years) of low-value surgeries by hospital districts in Finland in 2020–2021, divided by surgery, separately by public and private sectors. For name of hospital districts, see Figure 2 and Table 2.

The most commonly performed low-value surgeries were acromioplasties and partial meniscectomies in East Savo public hospitals at 67 (CI 43–101) per 10^5^ person-years for both, and partial meniscectomies in private Southwest Hospital District 48 (CI 41–55, change +58%) per 10^5^ person-years (Figure 3, Table 3, see Appendix).

**Table 3:** Incidence of low-value care based on hospital district, divided into years 2006–2007 and 2020–2021 by hospital type (private vs public)

Procedure Hospital district ^a^	Private hospitals		Change (%)	Public hospitals		Change
	2006–2007	2020–2021		2006–2007	2020–2021	(%)
Acromioplasty						
1	14 (9.0–20)	2.4 (0.79–5.7)	–82	70 (58–82)	–	–100
2	–	–	–	120 (90–150)	19 (9.7–34)	–83
3	–	2.9 (0.07–16)	–	280 (230–340)	67 (43–100)	–76
4	26 (23–29)	2.9 (2.1–4)	–89	83 (77–88)	4.5 (3.5–5.8)	–95
5	–	–	–	91 (69–120)	33 (20–51)	–64
6	9.3 (4.9–16)	–	–100	110 (92–130)	13 (7.9–21)	–88
7	0.7 (0.02–3.9)	1.8 (0.31–5.9)	160	100 (88–120)	NA	–
8	–	1.5 (0.11–6.6)	–	130 (110–160)	1.0 (0.03–5.7)	–99
9	–	–	–	240 (200–290)	20 (9.2–37)	–92
10	36 (26–47)	–	–100	180 (160–210)	28 (20–39)	–85
11	44 (36–52)	2.8 (1.3–5.4)	–94	77 (67–87)	11 (7.9–16)	–85
12	16 (11–23)	4.0 (1.7–7.8)	–76	230 (210–250)	1.5 (0.31–4.4)	–99
13	31 (26–37)	2.8 (1.5–4.9)	–91	95 (85–100)	0.45 (0.06–1.6)	–100
14	1.5 (0.25–4.7)	0.29 (0.00–2.7)	–80	89 (75–100)	11 (6.8–18)	–87
15	11 (6.7–17)	0.85 (0.06–3.6)	–92	100 (90–120)	3.1 (1.1–7)	–97
16	9.6 (4.7–17)	–	–100	180 (150–200)	25 (16–36)	–86
17	11 (6.4–17)	0.97 (0.07–4.2)	–91	100 (85–120)	3.9 (1.4–8.5)	–96
18	–	–	–	120 (100–150)	6.7 (2.3–15)	–95
19	30 (25–37)	9.9 (7–13)	–67	150 (140–170)	1.4 (0.48–3.1)	–99
20	7.0 (3.2–13)	1.9 (0.31–5.9)	–74	52 (41–67)	3.7 (1.2–8.6)	–93
Ankle arthroscopy						
1	–	–	–	1.6 (0.32–4.5)	2.4 (0.79–5.7)	57
2	–	–		3.4 (0.41–12)	–	
3	–	–	–	5.2 (0.63–19)	2.9 (0.07–16)	–44
4	1.3 (0.72–2.1)	0.22 (0.05–0.64)	–83	1.3 (0.69–2.1)	0.4 (0.14–0.9)	–68
5	–	–		1.5 (0.04–8.6)	–	
6	–	–	–	4.4 (1.6–9.6)	1.8 (0.3–5.7)	–59
7	–	–		1.4 (0.17–5.0)	–	
8	–	–		2.6 (0.44–8.4)	–	
9	–	–		–	–	
10	–	0.37 (0.00–3.4)	–	0.73 (0.02–4.0)	1.1 (0.079–4.7)	51
11	–	0.78 (0.13–2.5)	–	1.6 (0.47–3.8)	0.16 (0.00–1.5)	–90
12	–	–	–	2.3 (0.68–5.5)	1.7 (0.42–4.7)	–23
13	0.13 (0.00–1.2)	0.23 (0.01–1.3)	81	1.4 (0.48–3.1)	0.45 (0.06–1.6)	–67
14	–	0.58 (0.02–3.2)	–	1.8 (0.37–5.2)	–	–100
15	–	–	–	1.1 (0.13–3.9)	1.4 (0.23–4.5)	29
16	–	–	–	2.3 (0.38–7.3)	2.8 (0.58–8.2)	23
17	–	–	–	2.6 (0.7–6.6)	1.9 (0.4–5.7)	–24
18	–	–		2.3 (0.28–8.2)	–	
19	0.4 (0.03–1.7)	–	–100	0.94 (0.23–2.6)	0.5 (0.06–1.8)	–47
20	–	–		1.6 (0.19–5.7)	–	
Distal radius fracture						
1	–	–	–	3.6 (1.5–7.5)	10 (6.5–16)	190
2	–	–	–	1.7 (0.04–9.4)	24 (13–40)	1,300
3	–	–		–	22 (9.2–44)	
4	0.043 (0.00–0.40)	1.9 (1.2–2.8)	4300	4.3 (3.2–5.7)	9.6 (8.1–11)	120
5	–	–	–	2.3 (0.17–9.9)	13 (5.8–26)	480
6	–	–		–	20 (13–29)	
7	–	1.5 (0.18–5.3)	–	1.7 (0.29–5.6)	–	–100
8	–	–	–	5.3 (1.7–12)	11 (5.3–20)	110
9	–	–	–	5.8 (1.2–17)	15 (5.8–30)	150
10	–	–	–	4.4 (1.6–9.5)	12 (7.0–20)	180
11	–	2 (0.79–4.3)	–	7.4 (4.6–11)	19 (14–24)	150
12	–	0.99 (0.12–3.6)	–	1.5 (0.31–4.4)	15 (10–22)	920
13	0.13 (0.00–1.2)	0.23 (0.01–1.3)	80	4.6 (2.8–7.3)	7 (4.8–9.9)	51
14	–	0.29 (0.00–2.7)	–	2.1 (0.5–5.6)	19 (13–27)	840
15	–	0.28 (0.00–2.6)	–	1.1 (0.13–3.9)	17 (11–24)	1,400
16	–	–	–	2.7 (0.57–8)	11 (5.8–20)	310
17	–	–	–	0.64 (0.016–3.6)	17 (11–25)	2,500
18	–	–	–	1.7 (0.12–7.3)	20 (12–32)	1,100
19	–	1.6 (0.63–3.4)	–	6.3 (4.0–9.5)	9.4 (6.6–13)	48
20	–	–	–	5.1 (2.0–11)	5.6 (2.3–11)	9.1
Partial meniscectomy						
1	28 (21–36)	8.1 (4.6–13)	–71	260 (240–290)	9.5 (5.8–15)	–96
2	–	–	–	260 (220–310)	38 (24–58)	–85
3	–	16 (5.6–36)	–	210 (170–260)	67 (43–100)	–68
4	82 (77–87)	29 (26–32)	–64	130 (120–140)	12 (10–14)	–91
5	–	–	–	170 (140–200)	18 (8.6–32)	–89
6	24 (17–34)	20 (13–29)	–17	250 (220–280)	22 (15–31)	–91
7	9.1 (4.8–16)	27 (19–37)	200	120 (110–140)	–	–100
8	–	10 (4.9–19)	–	200 (170–230)	7.2 (2.9–15)	–96
9	–	–	–	120 (94–160)	21 (9.9–38)	–83
10	36 (26–47)	8.8 (4.5–15)	–75	130 (110–150)	34 (25–46)	–73
11	75 (65–86)	19 (15–24)	–75	150 (140–170)	23 (18–28)	–85
12	29 (22–38)	27 (20–35)	–6.9	170 (160–190)	14 (9.2–20)	–92
13	130 (120–150)	20 (16–25)	–85	180 (170–200)	5.4 (3.5–8.1)	–97
14	43 (34–54)	13 (8–19)	–70	160 (150–190)	27 (19–36)	–84
15	54 (44–66)	6.8 (3.5–12)	–88	150 (130–160)	13 (8.2–19)	–91
16	62 (48–79)	–	–100	140 (120–160)	11 (5.5–19)	–92
17	24 (17–33)	28 (20–38)	16	190 (170–210)	20 (13–28)	–90
18	0.57 (0.00–5.3)	–	–100	160 (140–190)	27 (17–41)	–83
19	110 (100–120)	48 (41–55)	–58	230 (220–250)	7.4 (5–11)	–97
20	32 (23–44)	26 (18–36)	–20	75 (61–92)	23 (16–33)	–69
Rotator cuff repair						
1	2.1 (0.56–5.3)	3.2 (1.2–6.7)	53	5.4 (2.7–9.9)	2.7 (0.93–6)	–51
2	–	–	–	18 (8.6–32)	5 (1.0–15)	–72
3	–	4.4 (0.32–19)	–	26 (12–48)	32 (16–58)	25
4	6.3 (4.9–7.9)	8.4 (7.0–10)	35	6.7 (5.3–8.4)	2 (1.3–2.9)	–71
5	–	–	–	5.4 (1.3–15)	3.4 (0.41–12)	–38
6	–	2.2 (0.44–6.3)	–	5.2 (2.1–11)	9.7 (5.2–16)	87
7	–	2.2 (0.45–6.4)	–	14 (8.8–22)	–	–100
8	–	2.1 (0.25–7.5)	–	14 (7.3–23)	2.6 (0.43–8.3)	–81
9	–	–	–	2.9 (0.21–12)	6.2 (1.3–18)	120
10	5.4 (2.3–11)	3.3 (0.99–8)	–39	16 (9.7–24)	16 (10–25)	5.7
11	3.8 (1.9–6.8)	5.8 (3.5–9.1)	53	6 (3.6–9.6)	5.8 (3.5–9.1)	–3.9
12	4.3 (1.9–8.2)	5.7 (2.9–10)	34	27 (20–35)	6.2 (3.3–11)	–77
13	2.6 (1.3–4.8)	3.4 (1.9–5.6)	29	5.4 (3.4–8.2)	1.7 (0.71–3.4)	–69
14	2.4 (0.65–6.1)	4.6 (2.0–9.1)	95	10 (5.9–16)	9.8 (5.7–16)	–2.3
15	1.1 (0.13–3.9)	2.3 (0.61–5.8)	110	9.3 (5.4–15)	7.6 (4.1–13)	–18
16	3.7 (1.0–9.4)	–	–100	18 (11–28)	14 (7.5–23)	–24
17	2.6 (0.7–6.6)	5.2 (2.2–10)	100	6.4 (3.1–12)	12 (6.9–18)	82
18	–	–	–	23 (14–36)	4.9 (1.3–12)	–79
19	8.8 (6.0–12)	14 (10–18)	57	12 (8.5–16)	9 (6.3–12)	–23
20	1.2 (0.08–5.0)	3 (0.81–7.6)	150	2.7 (0.66–7.4)	3 (0.81–7.6)	8.1
Wrist arthroscopy						
1	0.26 (0.00–2.4)	0.24 (0.00–2.3)	–5.7	3.9 (1.6–7.8)	0.24 (0.00–2.3)	–94
2	–	–		–	1.7 (0.04–9.3)	–
3	–	–	–	7.8 (1.6–23)	4.4 (0.32–19)	–43
4	0.65 (0.27–1.3)	1.2 (0.69–1.9)	86	1.4 (0.79–2.2)	1.4 (0.83–2.2)	0.28
5	–	–	–	–	3.4 (0.41–12)	–
6	–	–	–	–	2.9 (0.78–7.3)	–
7	–	1.8 (0.31–5.9)	–	3.1 (0.94–7.7)	–	–100
8	–	1 (0.03–5.8)	–	1.1 (0.03–5.9)	–	–100
9	–	–	–	4.8 (0.8–15)	2.1 (0.05–11)	–57
10	1.1 (0.08–4.7)	1.1 (0.08–4.7)	1	5.1 (2.0–10)	14 (8.1–21)	170
11	2.6 (1.1–5.2)	2.2 (0.88–4.5)	–15	7.3 (4.5–11)	2.5 (1.1–4.9)	–65
12	–	0.99 (0.12–3.6)	–	1.5 (0.31–4.4)	5.2 (2.6–9.5)	250
13	0.75 (0.16–2.2)	0.23 (0.01–1.3)	–70	2.6 (1.3–4.8)	1.5 (0.57–3.1)	–44
14	0.59 (0.02–3.3)	–	–100	3 (0.96–6.9)	1.4 (0.24–4.6)	–51
15	1.6 (0.34–4.8)	2.3 (0.61–5.8)	38	0.27 (0.00–2.6)	–	–100
16	–	–		–	–	
17	–	–	–	0.96 (0.07–4.1)	0.97 (0.07–4.2)	1.2
18	–	–	–	–		
19	0.27 (0.01–1.5)	0.13 (0.00–1.2)	–54	–	0.75 (0.15–2.2)	–
20	–	–	–	–		

a For name of hospital districts, see Table 2. NA = not available.

### Factors describing the regional variation

**Figure 4:**
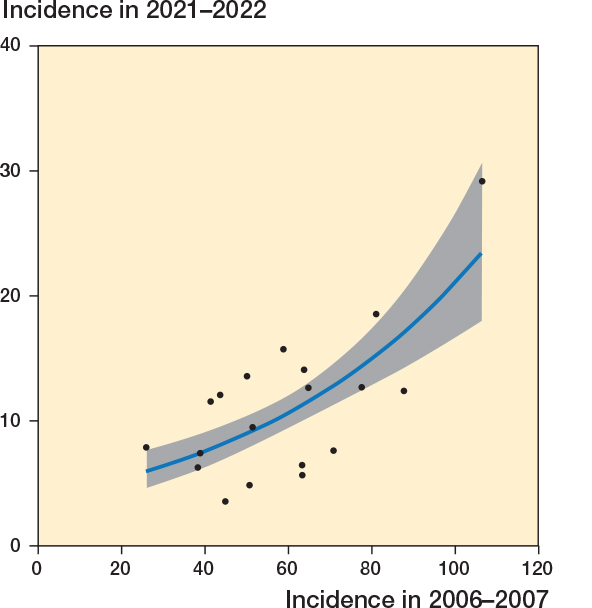
Correlation between the incidence in 2020–2021 and in 2006–2007 (r = 0.69, CI 0.37–0.87). The blue line with gray ribbon presents the Poisson regression model with 95% confidence intervals.

The incidence in years 2006–2007 had a positive correlation with the incidence in 2020–2021 (r = 0.69, CI 0.37–0.87; Figure 4). A Poisson regression model, adjusted for the mean population size, showed β = 0.015 (CI 0.01–0.02), indicating an increase in the incidence of low-value surgery with each unit increase in the incidence of reference years.

**Figure 5:**
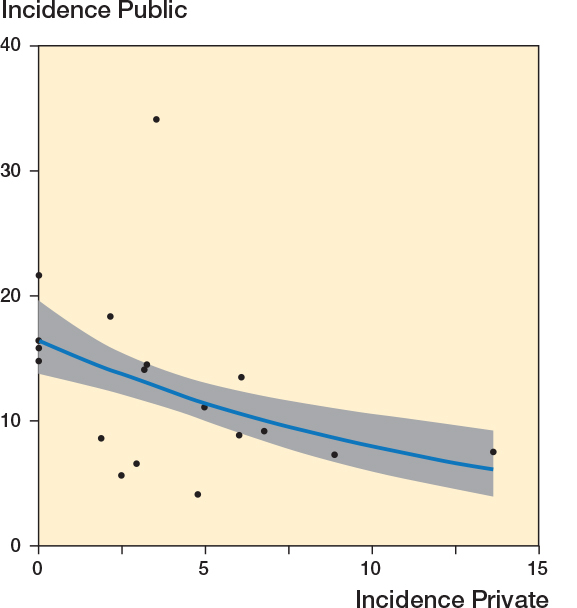
Correlation between the incidence of low-value surgeries in private and public hospitals in 2020–2021 (r = –0.43, CI –0.73 to –0.001). Also see legend to Figure 4.

There was a negative correlation between the incidence of private and public hospitals (r = –0.43, CI –0.73 to 0.001; Figure 5). A Poisson regression model, adjusted for the incidence in the reference years (2006–2007), showed β = –0.04 (CI –0.08 to 0.004), indicating a reduction in the incidence of low-value surgery with each unit increase in the incidence in private hospitals.

**Figure 6:**
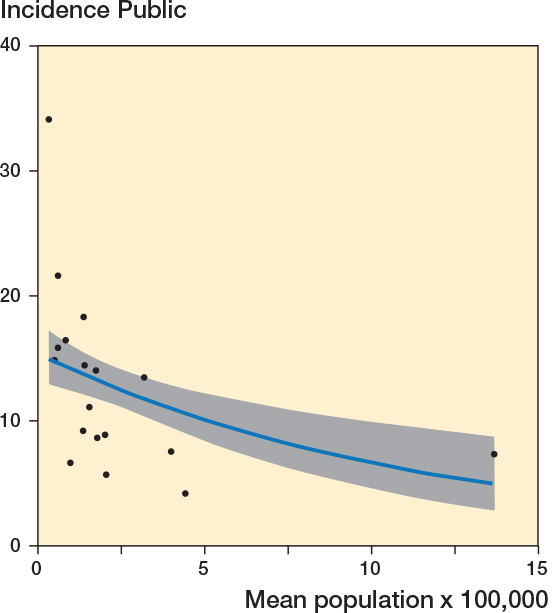
Correlation between the incidence of low-value surgeries in public hospitals and mean population size per region (r = –0.42, CI –0.72 to –0.02). Also see legend to Figure 4.

A negative correlation existed between the incidence in the public hospitals and mean population size (r = –0.42, CI –0.72 to 0.02; Figure 6). A Poisson regression model, adjusted for the incidence in the reference years (2006–2007), showed β = –0.04 (CI –0.09 to 0.01), indicating a reduction in the incidence of low-value surgery with each unit increase in mean population size.

## Discussion

We aimed to examine regional differences in common low-value musculoskeletal surgeries in Finland and to explore explanatory factors behind the variation. We found that the incidence of low-value surgery in Finland decreased greatly between 2006 and 2021 but great regional variation still exists, some districts performing up to 7 times more low-value surgeries compared with the lowest incidences. Such large variation is very unlikely to occur due to differences in morbidity and our analyses suggest that hospital treatment culture is the main determinant of low-value surgery; most low-value surgeries were performed in hospitals where these surgeries were historically more prevalent. Despite the uptake of new evidence, the incidences remained relatively high in 2020–2021 in many hospitals. Another factor associated with higher incidence was the size of the hospital: low-value care was more rampant in smaller hospitals suggesting that the uptake of evidence is slower in small non-academic hospitals. The hospitals with the lowest incidences of low-value surgeries are not necessarily the most evidence-based, yet our study suggests that these regions may be more aligned with current evidence-based guidelines [33].

The grassroots-level mechanisms behind the higher incidence cannot be explained with our data. It can be assumed that the regional variation in surgical procedures arises from a complex mix of factors tied to both clinical and socioeconomic contexts. Physicians’ beliefs regarding the efficacy and necessity of surgical interventions in specific clinical scenarios play an important role. Variations in these beliefs may be socially inherited from senior surgeons, and exposure to research can also vary, both shaping individual clinical judgment. Other possible factors are local patient preferences stemming from local treatment culture, dissemination of innovations, and the composition of the physician workforce [4]. The extent of regional variation observed was so substantial that it cannot be solely explained by patient-related factors, considering that clinicians ultimately make the treatment decision.

The downstream effects of diagnostic practices impact surgical decisions and increase overall healthcare costs [34]. For instance, the reimbursement policies for diagnostic tests such as those for degenerative tears influence the frequency of these diagnoses, potentially reducing the occurrence of low-value surgeries. Limiting initial diagnostic steps can prevent unnecessary procedures, as demonstrated by studies on MR imaging for knee pain. An RCT by van Oudenaarde et al. [35] found that MR imaging led to higher healthcare costs without improving outcomes, as it increases the referrals to orthopedic surgeons and therefore increases the incidence of arthroscopies in patients with traumatic knee symptoms.

Interventions aimed at reducing the use of diagnostic test procedures can thus play a crucial role in minimizing low-value care, as they prevent the cascade of further tests and procedures prompted by initial diagnostic findings. For example, a multifaceted quality improvement intervention aimed at hospitals and surgeons could help curb the overuse of diagnostic tests and subsequent unnecessary treatments [36].

### Strengths

This study was robust and comprehensive register data was of high quality, encompassing all surgeries conducted in both private and public hospitals across Finland. The inclusion of detailed information regarding the hospitals where surgeries were performed enabled us to accurately assess the incidence rates within specific hospital districts, enhancing the reliability of our findings.

### Limitations

This study placed sole reliance on operation and diagnosis codes. Consequently, some variation may be explained by coding errors; however, we believe errors occur mostly at random and systematic errors are unlikely to explain the observed variance. Furthermore, data based solely on coding limits our ability to provide comprehensive insights into the indications for surgeries. We considered only the primary procedure code, potentially overlooking additional procedures performed alongside the main one. Including the surgeries from population-wide data as low-value care based on the inclusion criteria of RCTs and meta-analyses can lead to including patients whose surgical indications fall outside the scope of evidence we considered. Nonetheless, this limitation is primarily relevant to distal radius fractures, where the current evidence is limited to only dorsally dislocated fractures, and thus other type of fractures (e.g., Smith, Barton, Chauffeur) cannot be explicitly categorized as low-value surgeries. Given the substantial volume of data analyzed and the rarity of these fractures, the incidence rates should be evenly distributed across all regions and therefore unlikely to significantly impact the study’s outcomes. In addition, COVID-19 may have reduced the incidences of elective surgical procedures, but we cannot measure the extent of its impact. However, the incidences remained significantly high in certain areas despite COVID-19, which highlights extent of the existing problem.

### Conclusion

The findings of this study reveal a notable disparity in the incidence rates of common low-value surgeries across different regions in Finland. The primary determinants contributing to this variation were historical incidence rates and the mean population size of the respective hospitals.

In perspective, a more comprehensive analysis that incorporates qualitative research methods, focusing on interviewing surgeons concerning their beliefs, research engagement, and financial motivations linked to epidemiological data, could enhance our understanding of the underlying mechanisms perpetuating the use of low-value surgical practices in certain hospitals.

## Notes

VP: conceptualization, methodology, formal analysis, investigation, and preparation of the original draft. AL, VM, TK: conceptualization, methodology, review & editing.

Handling co-editors: Cecilia Rogmark and Philippe Wagner

*Acta* thanks Stig Brorson and Rudolf W Poolman for help with peer review of this manuscript.
